# Competition for Cooperation: variability, benefits and heritability of relational wealth in hunter-gatherers

**DOI:** 10.1038/srep29120

**Published:** 2016-07-12

**Authors:** Nikhil Chaudhary, Gul Deniz Salali, James Thompson, Aude Rey, Pascale Gerbault, Edward Geoffrey Jedediah Stevenson, Mark Dyble, Abigail E. Page, Daniel Smith, Ruth Mace, Lucio Vinicius, Andrea Bamberg Migliano

**Affiliations:** 1 Department of Anthropology, University College London, London WC1H 0BW, United Kingdom; 2 Department of Genetics, Evolution and Environment, University College London, London WC1E 6BT, United Kingdom

## Abstract

Many defining human characteristics including theory of mind, culture and language relate to our sociality, and facilitate the formation and maintenance of cooperative relationships. Therefore, deciphering the context in which our sociality evolved is invaluable in understanding what makes us unique as a species. Much work has emphasised group-level competition, such as warfare, in moulding human cooperation and sociality. However, competition and cooperation also occur within groups; and inter-individual differences in sociality have reported fitness implications in numerous non-human taxa. Here we investigate whether differential access to cooperation (relational wealth) is likely to lead to variation in fitness at the individual level among BaYaka hunter-gatherers. Using economic gift games we find that relational wealth: a) displays individual-level variation; b) provides advantages in buffering food risk, and is positively associated with body mass index (BMI) and female fertility; c) is partially heritable. These results highlight that individual-level processes may have been fundamental in the extension of human cooperation beyond small units of related individuals, and in shaping our sociality. Additionally, the findings offer insight in to trends related to human sociality found from research in other fields such as psychology and epidemiology.

Many unique aspects of human sociality such as language, theory of mind and cultural norms have been proposed to provide the framework for human cooperative behaviour^1^,^2^,^3^,^4^, which stands alone in its scale and ubiquity between unrelated individuals^5^. Cooperation has been fundamental to the demographic success of our species - resource exchange, collective action and specialisation have increased our efficiency at surmounting a vast array of environmental pressures^6^,^7^. Therefore deciphering the context in which human cooperation and sociality evolved is invaluable to understanding what makes us unique as a species.

Inter-individual differences in sociality have been reported to have fitness implications in numerous taxa. For instance, in various non-human primates, greater social integration or social capital has been associated with increased longevity, offspring survival or mating access^8^,^9^,^10^. However, in humans, the link between individual differences in social integration and reproductive fitness has received little attention. There is substantial evidence that humans have a series of psychological and physiological reinforcement mechanisms encouraging the formation and maintenance of social relationships^11^. The existence of these proximate mechanisms encouraging social integration, implies social ties must also have some ultimate fitness enhancing function within our species. We pay particular attention to the cooperative function of human social relationships, and investigate the importance of inter-individual differences in relational wealth^12^ (access to cooperation from group members) within the group.

Substantial research into the evolution of human sociality and cooperation has focused on group-level explanations. Such explanations emphasise the importance of cooperation between unrelated individuals in large scale warfare and resource competition between groups^13^. These theories assert that human sociality includes a suite of traits such as tendencies to form in-group biases and internalise cultural norms, which evolved to help individuals function in highly cooperative groups^4^. However, competition and cooperation can also occur at the individual level between members of the same group; and as alluded to above, in numerous non-human taxa differences in sociality within the group have important implications for fitness. Therefore, if certain individuals are better able to accrue cooperative social relationships with others (relational wealth), differences in fitness at the individual level may emerge within groups. We hypothesise that individual level ‘competition for cooperation’ may have been an important driving force in human evolution and fundamental in shaping our sociality.

We attempt to identify the role of inter-individual variation in relational wealth in the dynamics of within-group competition among BaYaka hunter-gatherers. The BaYaka are simple and mobile hunter-gatherers - they consume food they forage soon after acquisition and lack storage mechanisms, and are also politically egalitarian; such societies are the best extant approximation of the ecological conditions under which our species evolved. Therefore, although the BaYaka are an extant population of cognitively modern humans, their forager lifestyle offers a valuable opportunity for inference regarding human evolutionary history.

Whereas in other subsistence modes food storage is an option, simple hunter-gatherers rely profoundly on food sharing to mitigate risks associated with the unpredictability of their foraging niche^14^,^15^; thus cooperation is at the heart of these populations. Although these societies are usually egalitarian^16^, social interaction and exchange is still structured within these populations^15^,^17^, and certain individuals may be better able to accrue cooperative links with others by means not dependent on formal hierarchy. In such a context, where individuals are so reliant on cooperative relationships, those with more relational wealth are likely to have an evolutionary advantage. Women may benefit from more access to allocare and provisioning, in turn increasing the health and survival prospects of their offspring, and aiding in the trade-off between childcare and foraging effort^18^. Men with more cooperative partners, may profit from biased resource allocation towards their families, and also have increased access to mates^19^. Therefore, in these societies where material wealth is absent, it may be relational wealth that drives documented patterns of individual fitness variance^20^. Indeed some evolutionary anthropologists have noted the likely relevance of the related concept of social capital to hunter-gatherers^21^. Social capital is traditionally used in economics and sociology, and the term has been used to describe social relationships and interactions with others that generate returns for the individual^22^,^23^. Kaplan *et al*. hypothesise that since activities such as food sharing are often not uniform in hunter-gatherer groups, markets for cooperative partners emerge and social capital is likely to become relevant for consumption patterns and fitness^21^.

Here we use economic gift games to construct and compare individual cooperative networks in three BaYaka camps. Our results demonstrate the presence of individual variation in relational wealth, which is particularly striking among men. We show that those with more relational wealth receive food transfers from a larger number of individuals than their peers, and this is reflected in their significantly higher BMI; women with more relational wealth also have significantly higher age-specific fertility. The data also suggest there is a heritable component to relational wealth, and that cooperative alliances may be transmitted inter-generationally. While cooperation may have been important for increasing group resilience in warfare and resource competition, our findings indicate that individual level competition for cooperation within the group may also have been fundamental in shaping human sociality.

## Results

### Individual variation in relational wealth

**Figure 1:**
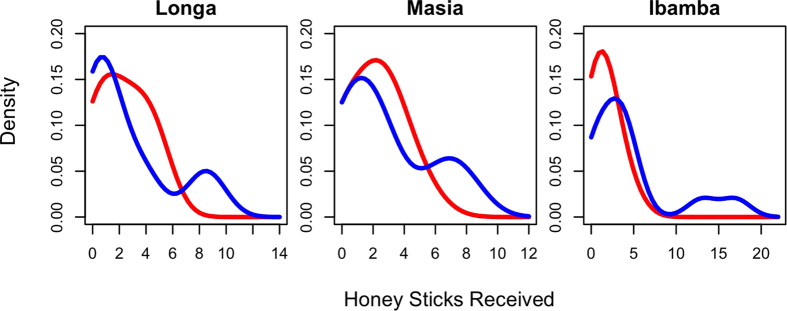
The distribution of relational wealth. Kernel-Density Distributions of the number of honey-stick nominations received per individual for men (blue) and women (red) in three Mbendjele camps. Camp names are indicated above each graph.

We constructed an adult-to-adult gift network by playing a honey stick gift game (HSGG)^24^ with all adults in three BaYaka camps (n = 97, 52 female), where each participant must choose the distribution of three honey sticks amongst other members of his/her camp. Figure 1 shows the distributions of total number of honey sticks received by an individual in the HSGG, which is our measure of relational wealth. It is clear that there is individual-level variation in number of gifts received for both sexes. It is noteworthy that the effect of individual differences in genetic relatedness to members of one’s camp on number of gifts received does not reach significance (p = 0.067; R^2^ = 0.036) (see Supplementary Table 1). The extent of male variation is particularly striking producing multi-modal distributions in all three camps, where certain men receive substantially more honey sticks than their peers. Levene’s tests highlight a significantly larger variance in male than female relational wealth in two of the three camps – Longa (p = 0.023; n = 47, 25 female) and Ibamba (p = 0.011; n = 30, 18 female) (see Supplementary Table 2). The lack of significance in camp Masia (p = 0.123; n = 20, 9 female) is likely a result of the small sample that is concomitant with the camp size.

This trend may reflect the fact that usually male hunting production is both more variable and shared more widely than female gathered foods in hunter-gatherer socities^25^,^26^, thus there are likely to be larger differences between men in opportunities to form alliances via food sharing. In fact, acquisition of social benefits has been postulated as the driving force behind male specialisation in foraging for unpredictable resources that are widely shared^27^. Additionally, a central aspect of Mbendjele life is the process of undergoing sex-specific initiation rites in order to gain membership to various religious cults, which increase bonding and solidarity amongst members; there is greater variation in membership to these religious cults amongst men. Nevertheless there is substantial variation in gifts received by both sexes, suggesting that if these relationships translate to benefits related to survival or reproduction, both men and women with more relational wealth can gain advantage over fellow camp members.

### Relational wealth variation results in individual differences in overcoming environmental risk, and is associated with higher female fertility

**Figure 2:**
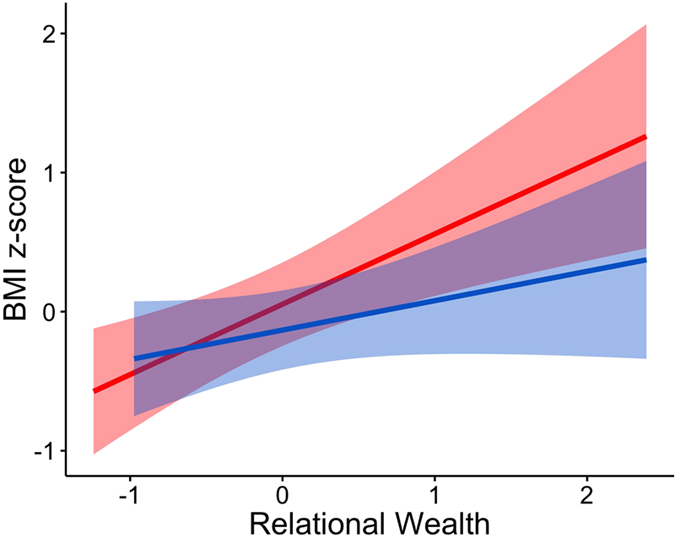
Relational wealth and body mass index (BMI). Relationship between relational wealth and BMI z-score (standardised by sex and age category–pre/post reproductive age for women and over/under 45 for men). Shaded bands indicate 95% confidence intervals. Blue line and shaded band represent males, red line and shaded band represent females.

Using multiple regression we found a significant association between our measure of relational wealth (HSGG nominations) and the number of camp members from whom an individual receives food in real world transfers (β = 0.24; p = 0.005; n = 53) (see Supplementary Table 3 for full results). We also find a significant relationship between BMI and relational wealth for both men (β = 0.53; p = 0.032; n = 39) and women (β = 0.90; p = 0.003; n = 34) (see Fig. 2; see Supplementary Table 4 for full results). Hunter-gatherer subsistence is highly unpredictable, thus food transfers between households are vital in buffering this high acquisition risk^14^,^28^. Although these societies are often characterised by norms promoting widespread sharing^29^,^30^, research shows that food transfers are biased by kinship ties, reciprocal relationships and foraging effort of others^31^,^32^,^33^. The findings here indicate that those individuals with more relational wealth are better able to secure a stable nutritional income, and tackle this fundamental adaptive problem.

Maintaining a healthy body weight may also be particularly important for female fertility as it avoids secondary amenorrhea^34^. Indeed we find relational wealth is a significant predictor of female age-specific fertility (β = 0.19; p = 0.010; n = 49) (see Supplementary Table 5). The result cannot be explained by reverse causality i.e. HSGG participants preferably distributing their honey sticks to women who have more offspring and thus may be in greater need: Female participants were at different stages of their reproductive career and offspring of older participants may have already reached adulthood, thus a participant’s total fertility does not necessarily match their current number of dependent (under 16) offspring. We find no significant correlation between a woman’s current number of dependent offspring living in the household and relational wealth (G = 0.14, p = 0.280, n = 51), suggesting in the initial association between relational wealth and age-specific fertility relational wealth is affecting fertility rather than vice-versa. Nevertheless the result must be treated as preliminary since our measure of fertility and relational wealth reflect different timescales; specifically relational wealth is a measure reflecting one point in time (the data collection period), whereas fertility reflects the length of ego’s reproductive career thus far.

### Relational wealth is inherited from fathers

**Figure 3:**
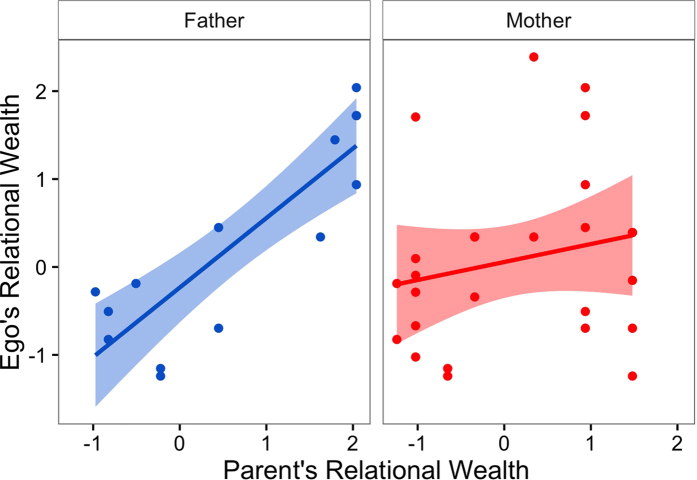
The inheritance of relational wealth. Scatter plots representing the relationship between ego’s relational wealth and ego’s father’s (blue) and ego’s mother’s (red) relational wealth. Shaded bands indicate 95% confidence intervals.

In order to test if relational wealth is heritable, we conducted gamma correlations between the number of honey stick nominations of parents and their adult offspring in the HSGG (see Fig. 2). Ego’s (male or female) relational wealth as an adult is positively correlated with ego’s father’s (G = 0.65, p = 0.002; n = 14), and ego’s mother’s (G = 0.17, p = 0.294; n = 26) relational wealth (see Fig. 3); but these results are only significant for the former. Although this hunter-gatherer society is egalitarian^29^,^35^,^36^–no individuals can exert any authority over others, and there are no hierarchical positions - the results here indicate there is a degree of heritability of relational wealth; we explore the potential mechanisms in the discussion.

## Discussion

We find that relational wealth varies by individual, provides health and fertility benefits and is partially heritable. These results highlight that in the absence of material wealth accumulation and social hierarchy, relational wealth may be an important determinant of individual fitness among simple hunter-gatherers. Individuals vary widely in their access to cooperation from fellow camp members, and those with more relational wealth are better equipped to overcome the high risk that characterises the hunter-gatherer lifestyle, since they have a significantly larger pool of food donors to insure against nutritional shortfalls. In addition to augmenting survival and health outcomes, social ties appear to increase reproductive rates of the BaYaka. Women with more relational wealth have higher age-specific fertility, a relationship which may be mediated by BMI since low body-weight disrupts ovulatory processes^34^. Additionally, we previously demonstrated that men with very high relational wealth are more likely to achieve polygyny in this group, which increases their reproductive rate^19^. Studies of other foraging societies have also reported positive associations between male social status and fertility, by examining the effect of hunting ability on mating access and reproductive outcomes^37^,^38^.

Egalitarian hunter-gatherers lack the heritable hierarchical positions which are found in agricultural and industrialised societies^16^, however, our results indicate partial heritability of relational wealth. We did not investigate the mechanism for this heritability explicitly, but there are several possibilities. Genetic factors have been shown to influence social network positioning in human and non-human primates^39^,^40^. Additionally, the inheritance may operate via the direct transmission of cooperative alliances from parents to offspring. This may explain the significant association with paternal but not maternal relational wealth - if relationships are transmitted inter-generationally, an individual’s relational wealth would be more closely associated with the parent whose sex has higher variability in number of social relationships. This inheritance of social ties would increase the evolutionary advantage of strengthening one’s social network since the associated benefits can accrue over multiple generations. Therefore, in hunter-gatherer groups which are often egalitarian and do not accumulate material resources, relational wealth may drive documented patterns of inter-individual fitness variance and fertility inheritance^20^,^41^, and may be the resource that is transmitted inter-generationally.

These findings offer a significant contribution to our understanding of human social evolution. The benefits of social bonds and importance of individual differences in social positioning have been identified for numerous taxa including non-human primates, feral horses and bottlenose dolphins^10^,^42^,^43^. Social ties have been associated with a variety of benefits in different species including increased longevity, offspring survival and mating access, enhanced dominance rank and reduced harassment^8^,^9^,^42^,^44^. However, similar research investigating the importance of inter-individual differences in sociality among humans is scant. This study differs from those in non-human taxa in its specific focus on cooperative networks (rather than proximity networks for example), nevertheless we still demonstrate that individual variation in an aspect of human sociality (relational wealth) has an important impact on health and fertility in hunter-gatherers.

Many investigations in to the evolution of human cooperation and sociality, specifically its widespread nature and extension beyond kin ties, have emphasised the importance of inter-group competition. These explanations highlight that human sociality evolved to facilitate group wide cooperation, since groups with cultural norms which are better able to promote cooperation and group beneficial behaviours outcompete others^4^. Our results do not undermine the possibility of selection at the group level, but draw attention to the importance of the role of cooperation in competition within the group. We find substantial inter-individual variability in access to cooperation (Fig. 1), which largely cannot be explained by kinship networks (Supplementary Table 1), and has meaningful consequences for health and fertility outcomes (Fig. 2/Supplementary Table 5). Cooperation is an integral means by which hunter-gatherers deal with their unpredictable environment, and extends across many activities including childcare, foraging and food sharing^17^,^45^,^46^. In the same way that groups with a greater capability to harness cooperation performed well in warfare and resource competition^4^, here we show that individuals within groups who harness more cooperation have increased resilience against the unpredictable foraging niche typifying hunter-gatherer subsistence.

Our findings suggest consideration of within-group competition is crucial to a complete understanding of the evolution of human sociality. These results indicate that over their evolutionary history some hunter-gatherer individuals may have outcompeted other members of their group by expanding their cooperative networks beyond the small close kin units ubiquitous in the animal kingdom. Therefore, although we may have psychological tendencies to form in-group biases and internalise cultural norms as a result of inter-group competition^4^,^47^, many of our derived social traits may also reflect within-group competition. Research from psychology and epidemiology on modern populations demonstrate a number of findings consistent with our results such as - positive associations between individual social integration and mental and physical health^48^; a psychological tendency for individuals to evaluate their social positioning relative to their peer group^49^; and neuroendocrine mechanisms encouraging the formation and maintenance of friendships^11^.

## Methods

This study has full approval from the Ethics Committee of University College London, and the methods were carried out in accordance with the approved guidelines. Informed consent was obtained from all participants and research permission granted by the Republic of Congo’s Ministry Of Scientific Research. The fieldwork took place between March and July 2014.

### Study population

Our study uses data from the Mbendjele BaYaka, a subgroup of the BaYaka who speak Mbendjele language and whose residence spans across the forests of Congo and Central African Republic. BaYaka subsistence techniques include hunting, trapping, fishing, gathering and honey collecting; as well as some trade with neighbouring farmer groups. Food sharing is an integral component of BaYaka subsistence and culture. The BaYaka live in *langos* - multi-family camps constituted of a number of *fumas* (huts) in which nuclear families reside; camp size tends to vary from 10–60 individuals, and genetic relatedness within camps is low^36^. The BaYaka are predominantly serially monogamous, with some incidence of polygyny^19^. We visited three camps in the Likoula and Sangha regions of Congo’s Ndoki Forest (see Supplementary Fig. 2 for map).

### HSGG

This game was played with all willing members of a camp and was completed as quickly as possible, usually within 2–3 days in each camp. All instructions were spoken in French by the researcher, and then immediately repeated in Mbendjele by the translator. The game was based on the procedure of Apicella *et al*. 24.The key features of our protocol for the game were:

1. Participants were asked to accompany the researcher and translator to a private area.
2. Participants were shown three honey sticks, and told that real honey was within each batton.
3. Participants were told they must decide to whom they would like us (the researchers) to give the honey sticks.
4. Participants were told they could give freely i.e. all three sticks to one individual or one stick to three different individuals etc.
5. Participants were told they could nominate any adult in their camp other than themselves.
6. After the games had been completed with all adults in camp, the honey sticks were distributed according to the results.

### Food Transfer Observations

Households were observed by JT over a series of two to four hour time blocks, with households observed for a total of 24 or 36 hours depending on the camp. Observations were evenly distributed between 6 am and 6 pm and spread over several days. During observation periods, a record was made of all food produced by a focal household. If division of resource packages occurred, all recipient households were identified. For all food cooked and consumed by the household, the type and amount of food were recorded and all those who ate the food were identified.

### Anthropometrics

We measured height and weight of all willing and non-pregnant adults in each camp in order to calculate BMI. Height was measured to the nearest mm using a Harpenden anthropometer, and weight using a Philipps mechanical scale.

### Analyses

In all analyses, relational wealth is calculated as the number of nominations received in the HSGG standardised by camp and sex.

We use multiple regression to analyse the relationship between relational wealth and number of food sharing donors. The response variable is the number of different camp members observed to share food with ego during food transfer observations. The predictor is ego’s relational wealth, and controls are ego’s sex, age (see Supplementary Information for details on calculation of age), and length of time ego was observed in the food transfer observations. In one camp, participants were observed for 24 hours and in the other two camps participants were observed for 36 hours; therefore we use a dummy variable to control for this.

We use multiple regression to analyse the relationship between relational wealth and BMI for each sex. We control for whether ego is post-reproductive (females)/over 45(males) since there is a significant decline in BMI for these age-groups in our sample. We also control for camp membership (categorical).

We use multiple regression to analyse the relationship between relational wealth and female fertility. We use age and age^2^ as controls to account for the quadratic relationship between age and fertility. To check whether reverse causality may explain the significant association found, we conduct a gamma correlation between female relational wealth and the number of dependent offspring in their household. The gamma correlation is conducted using the *rococo* package in R; and is selected as it is appropriate for variables which contain many ties, such as number of dependent offspring.

For correlation analyses of ego’s and ego’s parents’ relational wealth we also use gamma correlations because they are appropriate for small sample sizes and data with ties.

## Additional Information

**How to cite this article**: Chaudhary, N. *et al*. Competition for Cooperation: variability, benefits and heritability of relational wealth in hunter-gatherers. *Sci. Rep.* **6**, 29120; doi: 10.1038/srep29120 (2016).

## Supplementary Material

Supplementary Information

Supplementary Dataset 1
