# Supplementary material for: Competition for Cooperation: variability, benefits and heritability of relational wealth in hunter-gatherers
Source: Sci Rep. 2016 Jul 12;6:29120. doi: 10.1038/srep29120 (PMC4941516; doi:10.1038/srep29120)
Supplement: Supplementary Information [file srep29120-s1.pdf]

Competition for Cooperation: variability, benefits and heritability of relational wealth in hunter-gatherers: Supplementary Information

Nikhil Chaudhary*; Gul Deniz Salali; James Thompson; Aude Rey; Pascale Gerbault; Edward Stevenson; Mark Dyble; Abigail Page; Daniel Smith; Ruth Mace; Lucio Vinicius; Andrea Bamberg Migliano.

Analysing the extent to which variation in the HSGG can be explained by relatedness to camp

In order to identify the degree to which variation in relational wealth could be explained by relatedness to camp members i.e. other players of the honey stick gift game, we calculated a relatedness score for all individuals. To do this, we first calculated total relatedness to ego's camp by adding up genetic relatedness between ego and all other camp members. For instance if ego has one sibling and one cousin in camp and is unrelated to all other camp members, his total relatedness would be 0.5 + 0.125 = 0.625. Data on genetic relatedness was known from conducting reproductive histories and genealogical interviews with all participants.

Having calculated total relatedness for all individuals, relatedness scores were attributed by standardising total relatedness by camp and sex in order to match how relational wealth scores were calculated - by standardising number of honey stick nominations by camp and sex.

We then conducted a linear regression of the effect of relatedness score on relational wealth, which indicates the effect of differences in relatedness to other camp members on relational wealth does not actually reach significance.

Supplementary Table 1: Regression analysing the effect of relatedness to camp on relational wealth.

Predictor	*Coeff.*	*p*
Relatedness score	0.19	0.067
$R^2 = 0.036$		

Supplementary Figure 1:

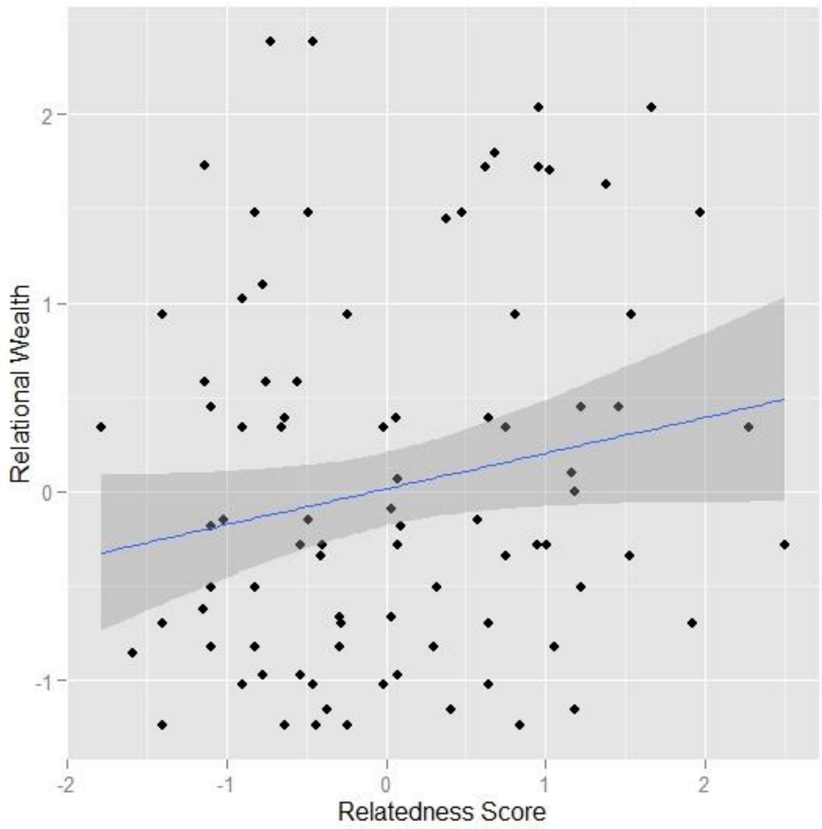

Supplementary Figure 1: Scatter plot of the relationship between relatedness score and relational wealth. Shaded bar indicates 95% confidence interval.

<u>**Male Vs female variation in honey stick nominations**</u>

Supplementary Table 2: **Male Vs Female variation in honey stick nominations**

Camp	n	Range	T	p
Longa				
Men	22	0-9	5.55	0.023
Women	25	0-5		
Ibamba				
Men	12	0-17	7.41	0.011
Women	18	0-5		
Masia				
Men	11	0-8	2.62	0.123
Women	9	0-5		

For each camp the following are indicated: Number of men and women; the range in number of honey stick nominations for each sex; and the test statistic (T) and p-value from Levene's tests examining whether there are significant differences between the sexes in variance of number of nominations. The Levene's tests indicate that in two of the three camps (Longa and Ibamba), there is significantly more variance in number of gifts received by men compared to women; the lack of significance in Masia is likely to be partially due to small sample size.

Results of analysis of relational wealth and food sharing donors

Supplementary Table 3: Results from multiple regression analysis of the effect of ego's relational wealth on the number of individuals observed to share food with ego during food transfer observations.

Number of food sharing donors; n=53

Predictor	Coeff.	p
Relational wealth	0.24	0.005
36h Observation	0.89	0.000
Male	-0.19	0.297
Age	-0.00	0.657

Some individuals' food transfers were observed for 36 hours and other only for 24 hours; therefore, the variable '36h Observation' is a dummy variable used to control for whether ego's food transfers were observed for 36 hours rather than the reference category of 24 hours. We also use a dummy variable to control for ego's sex, where the reference category is female; and a continuous variable to control for age. We find that our measure of relational wealth (honey stick nominations received) is strongly associated with number of donors in real life food transfers.

Results of analysis of relational wealth and Body Mass Index (BMI)

Supplementary Table 4: Results from regression analysis examining the effect of relational wealth on a) female and b) male BMI.

	Female BMI, n=34		Male BMI, n=39	
Predictor	***Coeff.***	***p***	***Coeff.***	***p***
Relational wealth	0.90	0.003	0.53	0.032
Age-Group 2	-1.73	0.007	-1.45	0.011
Camp-Ibamba	-0.78	0.292	1.59	0.021
Camp-Longa	-1.07	0.162	-0.77	0.211

We control for whether an individual is post-reproductive (female)/over 45 (male) since there is a significant decline in BMI for these age-groups in our sample. Individuals over these age thresholds are denoted as part of 'Age-Group 2', compared to a reference category which includes individuals under these age thresholds. Both regressions also include camp membership as a control, the reference camp is Masia. We find a significant positive effect of relational wealth on BMI for both sexes.

Result of analysis of relational wealth and fertility

Supplementary Table 5: Results from regression analysis examining the effect of relational wealth on female age-specific fertility.

Female Fertility; n=49

Predictor	***Coeff.***	***p***
Relational Wealth	0.187	0.010
Age	0.146	0.000
Age^2	-0.001	0.000

We control for age and age^2 since there is a quadratic relationship between fertility and age. We find a significant positive association between relational wealth and female fertility.

Map of Camps

Supplementary Figure 2:

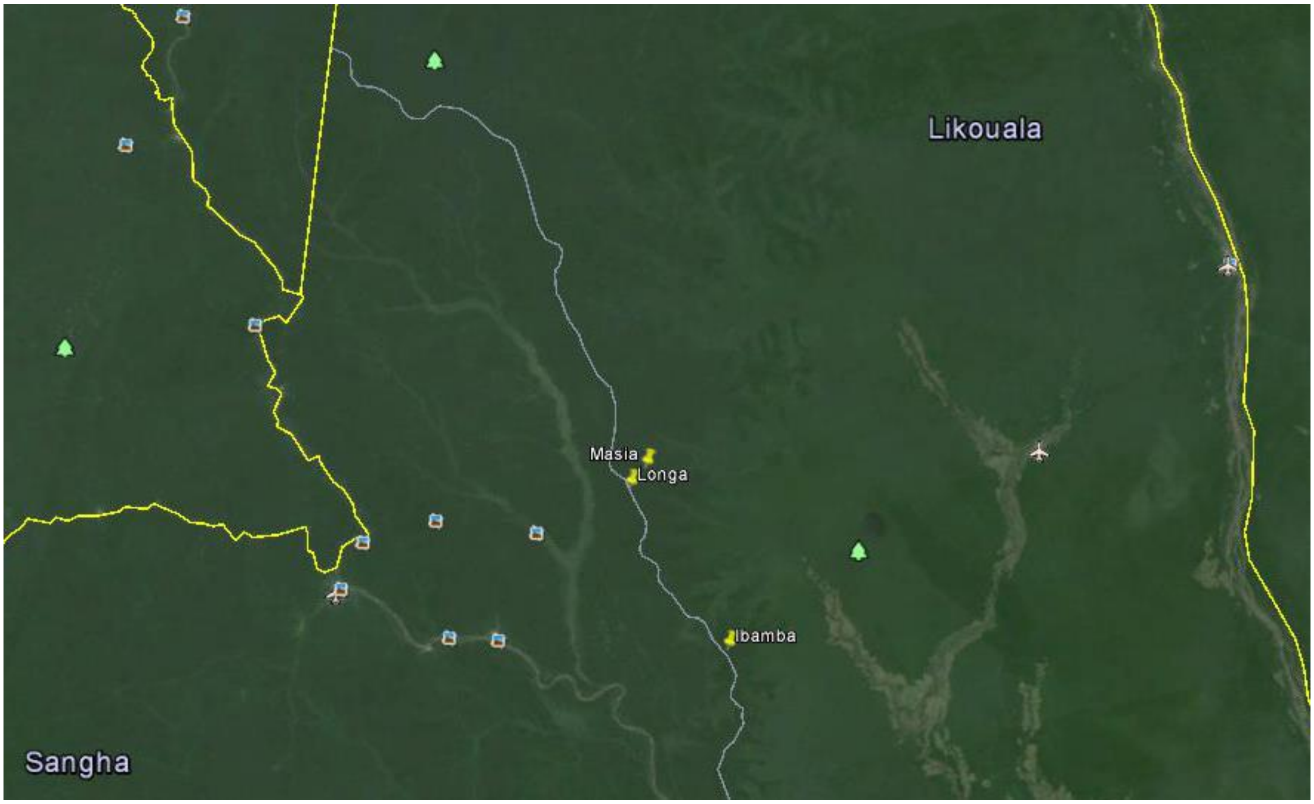

Supplementary Figure 2: Map of the three camps in study sample – Masia, Longa and Ibamba. Map created by NC in Google Earth, data from: Google, US Dept of State Geographer, Landsat. https://www.google.co.uk/intl/en_uk/earth/.

<u>Ageing the participants</u>

<u>*a) Constructing Relative Age Lists*</u>

In order to construct a relative age list photographs of all camp members were taken in each camp we visited. These were then divided in to rough age cohorts (<1; 1-2; 2-5; 5-10; 10-15; 15-20; 20-25; 25-30; 30-40; 40-60; 60+) based on visual estimations of the research team and our translators/guides. Following this, we called all members of a camp to sit down around a mat upon which we laid out all the photographs of a given cohort (starting from the youngest cohort). Firstly, we asked the participants to identify the oldest member of the cohort, and then we picked up the remaining photographs. Then one

by one we displayed the photographs of the other cohort members to the participants. After each photograph was displayed, we asked the participants the name of the individual on the photograph to verify they recognised who it was. The Mbendjele BaYaka are very mobile, and all the camps we visited were along the same long road; therefore all participants were very familiar with all members of our study population, including those from other camps. Next we asked where the photo should be placed on the mat – to the right of an already placed photo if the current individual was older, and to the left if s/he was younger. After each placement we confirmed that the newly placed individual was older than the photograph on its left and younger than that on its right; in some instances where individuals were considered equal in age, their photographs were placed above/below each other. If there was any disagreement among the participants, they were instructed to discuss amongst themselves and arrive at one answer together. After ranking within a cohort had finished, using the same process we checked whether individuals judged to be the youngest of that cohort were older/younger than the oldest individuals of the previous cohort. This process was repeated for all cohorts.

Our research was conducted over two fieldwork trips, and we obtained four relative age lists. At the end of the first trip, having obtained photographs of all individuals from this trip, we conducted the above process in two camps. This provided two distinct relative age lists, which both included all members of the study population we had met in this first fieldwork session. Then at the end of the second trip we obtained one more relative age list including all members of the entire study population from the minganga region (which includes Masia and Longa from the current study). Therefore we had three relative age lists (two partial and one complete) for the study population from minganga. Ibamba is the most isolated camp and is not in the minganga region. The Mbendjele in this camp were not familiar with members of the other camps, and vice versa. Therefore, using the same method we created a distinct relative age list for this camp.

b) Assigning initial age estimates

Unfortunately no one in our study population knew their own ages or ages of their offspring. We were able to assign absolute ages to a handful of participants. These individuals were either born during the study period, or confirmed by their parents to have been born at the same time as the road by the camps was constructed, which we know occurred in 1997. For all other participants, the three field researchers who visited all three BaYaka camps (NC, GS & JT) estimated a lower and upper bound of their age. These age ranges were informed by dental development, reproductive histories, birth order and visual appearance.

c) Deriving estimates

Finally, we derive a probability density distribution for ages of each individual by integrating the information from the age ranges and the ranked lists in a Gibbs sampling framework. Following this, we collapse the full distributions and generate age point estimates, namely the mean age for each individual.
